# Hemophagocytic lymphohistiocytosis as a rare complication of dengue haemorrhagic fever: a case report

**DOI:** 10.1186/s13256-023-03967-1

**Published:** 2023-06-01

**Authors:** Chamila Pradeep, Parackrama Karunathilake, Shamali Abeyagunawardena, Udaya Ralapanawa, Thilak Jayalath

**Affiliations:** 1 Teaching Hospital, Peradeniya, Sri Lanka; 2 Department of Medicine, Faculty of Medicine, University of Peradeniya, Peradeniya, Sri Lanka

**Keywords:** Hemophagocytic lymphohistiocytosis, Dengue, Cytopenia, Dexamethasone

## Abstract

**Background:**

Haemophagocytic lymphohistiocytosis (HLH) is an uncommon systemic inflammatory syndrome that can happen secondary to numerous conditions. It rarely occurs due to dengue infection causing significant mortality and morbidity even with appropriate treatment. The outcome is further poor if the diagnosis of HLH is delayed or left untreated. Therefore, a high degree of clinical suspicion is paramount in diagnosing HLH.

**Case presentation:**

A 17-year-old Sinhalese boy was admitted to a tertiary care hospital in Sri Lanka with a 4-day history of fever, headache, nausea, vomiting, and diarrhea. He was hemodynamically stable, and the serological investigation confirmed a dengue infection. On the fifth day of fever, he entered the critical phase of dengue infection, confirmed by ultrasound evidence of plasma leaking. However, he had ongoing high fever spikes during the critical phase, and even after the critical phase was over, the fever spikes continued. Simultaneously, hepatosplenomegaly was noticed, and he showed persistent thrombocytopenia, neutropenia, and anemia despite the resolution of the critical phase. Further, the workup revealed a serum ferritin level of > 3000 ng/mL triglyceride level of 314 mg/dL, and the bone marrow biopsy revealed an increased haemophagocytic activity. Secondary HLH was diagnosed on the basis of criteria used in the HLH-2004 trial and successfully managed with intravenous dexamethasone 10 mg/body surface area/day for the first 2 weeks, followed by a tapering regimen over 8 weeks.

**Conclusion:**

This case emphasizes the need to consider HLH as a potential complication when persistent fever and cytopenias are present after recovering from dengue fever, particularly in patients with unusual clinical features like hepatosplenomegaly. Early recognition and prompt treatment with appropriate immunosuppressive therapy, such as intravenous dexamethasone, can lead to a successful response and good prognosis.

## Background

Haemophagocytic lymphohistiocytosis (HLH), also known as hemophagocytic syndrome, is an uncommon systemic inflammatory clinical syndrome associated with numerous conditions [1]. HLH may be inherited (primary) or secondary to severe infections, malignancies, or rheumatologic conditions [2]. HLH is sporadically seen in clinical practice and is a rare complication of dengue characterized by persistent fever, pancytopenia, hepatosplenomegaly, and increased serum ferritin level. The overlap in clinical features makes diagnosing HLH in a dengue patient difficult, necessitating a bone marrow examination [3]. HLH is associated with significant mortality and morbidity even with appropriate treatment, and the outcome is further poor if the diagnosis is delayed or left untreated [4]. Therefore, a high clinical suspicion is paramount in diagnosing HLH, especially in an atypical presentation of a possible medical condition [2]. Here we present a case of HLH in an adolescent, which occurred secondary to dengue hemorrhagic fever, which was successfully treated and recovered.

## Case presentation

A 17-year-old Sinhalese boy was admitted to a tertiary care hospital in Sri Lanka with a 4-day history of high-grade intermittent fever associated with nausea, a few episodes of vomiting, and watery diarrhea. He also had a severe frontal headache, retro-orbital pain, arthralgia, myalgia, and anorexia without any abdominal cramps or right hypochondriacal pain, and he denied any respiratory or urinary symptoms. The headache was not associated with photophobia or phonophobia, and he did not have any previous medical conditions. He was a non-smoker and non-alcoholic.

On admission, he was mildly dehydrated, the capillary refilling time was less than 2 seconds, and he was not pale or icteric. There was no lymphadenopathy. His pulse rate was 100 beats per minute with 110/70 mmHg blood pressure without any postural drop. All other aspects of the physical examination, including the cardiovascular, respiratory, abdominal, and neurological systems, were unremarkable.

Initial blood investigations revealed a positive dengue non-structural protein 1 (NS1) antigen. In the full blood count, he had a white cell count of 2.7 × 10^9^/L, neutrophils 1.9 × 10^9^/L and lymphocytes 0.6 × 10^9^/L, and a red blood cell count of 5.04 × 10^9^/µL. The hemoglobin level was 14.7 g/dL, with a hematocrit of 41.5% and the platelet count was 125 × 10^3^/µL. His C-reactive protein (CRP) level was 30.9 mg/L, the aspartate aminotransferase (AST) level was 1716 U/L, and the alanine aminotransferase (ALT) level was 822 U/L. The investigations summary is given in Table 1.

**Table 1:** Investigations summary

Investigation	Day 1	Day 2	Day 4	Day 14
Complete blood count				
White cell count (× 10^9^/L)	2.7	2.9	4.1	5.3
Red blood cell count (× 10^9^/µL)	5.04	5.12	3.21	4.96
Hemoglobin level (g/dL)	14.7	15.1	10.7	14.2
Haematocrit (%)	41.5	45.2	33.5	41.6
Platelet count	125	48	27	186
Liver enzymes				
AST (U/L)	1716	987	234	52
ALT (U/L)	822	432	112	45
Renal functions				
Serum creatinine	89.0		96.1	
Inflammatory markers				
C-reactive protein (mg/L)	30.9		85.2	
Urinalysis				
Pus cells	Nil		Nil	
Red cells	Nil		Nil	
Proteins	Nil		Trace	
Microbiology				
Blood culture	No growth		No growth	
Special biochemical investigations				
Serum ferritin level was (ng/mL)			> 3000	
Triglyceride (mg/dL)			314	

*AST* Aspartate aminotransferase,* ALT* Alanine aminotransferase

After the initial workup, he was diagnosed with dengue fever in the febrile phase, and dengue febrile phase monitoring was initiated. He was administered oral paracetamol 1 g on an as-needed basis to control his fever. The following day, an ultrasound scan revealed free fluid in the abdomen, which was evidence of leaking, giving the diagnosis of dengue hemorrhagic fever in the critical phase, and routine fluid management and monitoring were done. In typical dengue hemorrhagic fever, the fever spikes, leucopenia, and thrombocytopenia improve after the critical phase is over. However, in this patient, even after the critical phase was over, he had continuous high-grade fever with persistent thrombocytopenia and a gradual drop in the hemoglobin level.

His subsequent red blood cell count was 3.21 × 10^6^/µL, and the platelet count was 27 × 10^3^/µL. He had also developed mild to moderate hepatosplenomegaly. The blood picture revealed thrombocytopenia with giant platelets and features suggestive of HLH. His serum ferritin level was > 3000 ng/mL (10–300 ng/mL), triglycerides 314 mg/dL, and the bone marrow biopsy revealed features of significant haemophagocytic activity. Based on these investigation findings, he was diagnosed to have HLH.

Then he was started with intravenous dexamethasone 10 mg per body surface area (17 mg) for the first 2 weeks, gradually tailed off over 8 weeks, and subsequently converted to oral dexamethasone, and the patient experienced excellent recovery. The fever settled within 24 h after starting intravenous dexamethasone, doubling white cell and platelet count. He was discharged after a 2-week hospital stay and completely recovered while reviewing in the clinic after 8 weeks.

## Discussion

This report describes a case of a 17-year-old Sinhalese boy with dengue fever who progressed to the critical phase of the infection. Even after the critical phase, the patient had a continuous high-grade fever, persistent thrombocytopenia, a gradual drop in hemoglobin levels, and hepatosplenomegaly. The diagnosis of HLH was made based on increased ferritin and triglyceride levels and confirmed with a bone marrow biopsy. This case is unique in several aspects. This patient's age at the time of diagnosis is relatively older than most reported cases, where HLH has been more commonly observed in children under the age of 10 [5]. At the same time, the patient had a successful response to intravenous dexamethasone therapy for 2 weeks followed by an 8-week tapering regimen. This treatment approach differs from some of the other cases reported in the literature, where different immunosuppressive agents and/or hemopoietic stem cell transplants were used [6]. Therefore, this case highlights the effectiveness of dexamethasone in managing HLH associated with dengue infection and adds to the existing knowledge on the management of this rare and potentially fatal complication of dengue fever.

HLH is a rare, potentially fatal hyperinflammatory and haemophagocytic syndrome causing severe hypercytokinemia with excessive activation of lymphocytes and macrophages associated with numerous conditions [2, 7]. The disease is seen in all ages and has no predilection for race or sex [8]. There are two main types of HLH; primary or familial HLH associated with genetic predisposition and secondary or sporadic HLH associated with other medical conditions, including infective, autoimmune, and malignant conditions [5]. Nevertheless, the classification of genetic and acquired is more appropriate for HLH than the primary and secondary [1]. Acquired (secondary) HLH can occur in all age groups, although there are no published data on its incidence or age distribution [9]. The infections causing HLH are shown in Table 2 [1]. HLH is an uncommon manifestation in dengue, and the diagnosis of HLH is difficult in dengue due to the overlap of the clinical features [3, 10].

**Table 2:** Infectious causes of HLH [7]

Viral infections	Epstein Barr virus and other herpesviruses Human immunodeficiency virus (HIV) Adenovirus Dengue Hantavirus Cytomegalovirus Hepatitis A/B/C viruses Measles Mumps Rubella Parvovirus B19 Enterovirus Influenza virus
Bacterial infections	*Staphylococcus aureus* *Campylobacter* spp. *Mycoplasma* spp. *Chlamydia* spp. *Legionella* spp. *Salmonella typhi* *Rickettsia* spp. *Brucella* spp. *Ehrlichia* spp. *Borrelia burgdorferi* *Mycobacterium tuberculosis*
Fungal infections	*Candida* spp. *Cryptococcus* spp. *Pneumocystis* spp. *Histoplasma* spp. *Aspergillus* spp.
Parasitic infections	*Plasmodium falciparum* *Plasmodium vivax* *Toxoplasma* spp. *Leishmania* spp. *Strongyloides* spp.

Dengue fever is caused by the Dengue virus, which belongs to the family Flaviviridae, genus Flavivirus, and is transmitted to humans by Aedes mosquitoes, mainly Aedes aegypti [11]. The clinical spectrum of dengue viral infection includes undifferentiated fever, dengue fever (DF), dengue hemorrhagic fever (DHF), and expanded dengue syndrome or isolated organopathy [12]. DF is an acute febrile illness with severe headache, myalgia, arthralgia, and rashes associated with leucopenia and thrombocytopenia [11–13]. Those infected with the dengue virus, especially for the first time, having a simple fever indistinguishable from other viral infections is called undifferentiated fever [12, 14]. DHF is characterized by the acute onset of high fever associated with signs and symptoms similar to DF in the early febrile phase, and subsequent plasma leakage leading to a tendency to develop hypovolemic shock (dengue shock syndrome) [11, 12, 14]. Expanded dengue syndromes include cases that do not fall into either dengue shock syndrome or DHF, associated with atypical and unusual manifestations [15]. After the clinical suspicion, confirmation of dengue infection can be made by detecting the virus, viral nucleic acid, antigens, or antibodies or by combining these techniques [16]. The virus can be detected in the circulation system within the first 4 to 5 days by checking for NS1 antigen [12, 16]. IgM antibodies are detectable in 50% of patients by three to 5 days after the onset of illness, increasing to 80% by day 5 and 99% by day 10, where it peaks about 2 weeks after the onset of symptoms and then generally decline to undetectable levels over 2 to 3 months. Anti-dengue serum IgG is generally detectable at low titers at the end of the first week of illness, increases slowly after that, and is detectable in serum after several months, which may present even for a lifetime [16]. Our patient demonstrated classic symptoms of DF such as fever, severe frontal headache with retro-orbital pain, arthralgia, and myalgia, where the NS1 antigen positivity confirmed the diagnosis, facilitating further management.

Persistent fever following dengue infection may occur due to sepsis and expanded dengue syndrome, including HLH [17–19]. HLH is an unusual hematological manifestation of expanded dengue syndrome, whereas other manifestations include disseminated intravascular coagulopathy and cytopenias [15]. There are several reported cases of HLH as a secondary manifestation of dengue infection in both the pediatric and adult populations, where most have occurred in patients without any other comorbidities. Nevertheless, one case has reported co-infection with *Plasmodium vivax*, and another has occurred in a child with beta-thalassemia major [9, 19–26].

The pathogenesis of HLH was first thought to result from the inability to clear infections in immunodeficient patients [20]. However, HLH in immunocompetent patients disproved that theory later, and the identification of cytotoxic pathway mutations as the primary cause of genetic HLH has elucidated the mechanism of this disease [1]. All forms of HLH are thought to be due to impairment in the function of cytotoxic T lymphocytes and natural killer (NK) cells, associated with a potentially fatal cytokine storm and hyperferritinemia [1, 21]. However, the exact mechanism is less precise for the nongenetic forms of HLH [9]. The inability to clear the antigenic stimulus to turn off the inflammatory response leads to hypercytokinemia seen in HLH [22]. If antigen removal is inefficient, the inflammatory stimulus will not be terminated, resulting in a final common pathway of HLH with uncontrolled hypercytokinemia, sustained macrophage activation, and tissue infiltration [22–24].

The clinical features of HLH appear to be due to CD8+ T-cell expansion, activation and infiltration of visceral organs associated with macrophage activation, and the release of multiple cytokines and chemokines [2]. The initial symptoms of HLH are nonspecific and may overlap with other inflammatory or hematopoietic diseases, and the diagnosis of HLH is based on the diagnostic criteria as revised for HLH-2004 [5, 25]. According to HLH-2004, there are two main criteria; Criterion 1 and 2. The diagnosis of HLH can be established if Criterion 1 or 2 is fulfilled. Criterion 1 included a molecular diagnosis consistent with HLH. Criterion 2 included fulfilling five of the eight criteria, namely fever, splenomegaly, cytopenias (affecting 2 of 3 lineages in the peripheral blood, hemoglobin < 9 g/dL, platelets < 100 × 109/L, and neutrophils < 1.0 × 109/L), hypertriglyceridemia, or hypofibrinogenemia (fasting triglycerides ≥ 3.0 mmol/L (i.e., ≥ 265 mg/dL), fibrinogen ≤ 1.5 g/L). Hemophagocytosis in bone marrow or spleen, or lymph nodes, no evidence of malignancy, low or no NK cell activity (according to local laboratory reference), ferritin ≥ 500 mg/L, and sCD25 (i.e. Soluble IL-2 receptor) ≥ 2400 U/mL are also included in Criterion 2 [5]. Our patient developed continuous high-grade fever, thrombocytopenia, anemia, and mild to moderate hepatosplenomegaly. His serum ferritin and triglyceride levels were high, fulfilling the criteria for diagnosing HLH. However, the bone marrow biopsy was also done, although it was unnecessary since the diagnosis could already be made with available information. However, since cytopenias can occur in uncomplicated dengue infection, bone marrow examination is justifiable to confirm the diagnosis of HLH and exclude other possibilities like hematological malignancies [26]. Our patient’s bone marrow examination revealed significant haemophagocytic activity, which confirmed the diagnosis of HLH, and the treatments were started promptly.

The management principles of HLH include suppression of hyperinflammation, elimination of activated immune cells, elimination of triggers, supportive therapy (neutropenia, coagulopathy), and replacement of the defective immune system [1, 4, 21, 27]. Immediate suppression of severe hyperinflammation should be done to prevent life-threatening outcomes of HLH [1, 2, 9, 26]. The treatment protocol includes induction, salvage, and continuation therapies [2, 27]. Suppression of hyperinflammation and the elimination of activated immune cells can be achieved with corticosteroids, intravenous immunoglobulins, cyclosporin A, anti-cytokine agents like etoposide, and monoclonal antibodies such as alemtuzumab and rituximab [4, 5, 21]. Corticosteroids are the first choice to suppress hypercytokinemia. The first-line option is dexamethasone; since dexamethasone crosses the blood–brain barrier better than prednisolone, suppresses the central nervous system inflammation more effectively [9]. The 2004 treatment protocol developed at the second international meeting of the Histiocyte Society recommends an 8-week induction therapy with corticosteroids, etoposide, and cyclosporine A [1, 23]. Anti-infectious therapy can eliminate the trigger and replace the defective immune system with a hemopoietic stem cell transplant [4, 21]. Therefore, the HLH-2004 guideline recommends chemo-immunotherapy with etoposide, dexamethasone, cyclosporine A upfront, and intrathecal therapy with methotrexate in selected patients. Subsequent hematopoietic stem cell transplantation is recommended for patients with familial disease, with a proven molecular diagnosis, or with severe, persistent, or reactivated disease [5]. In patients with milder forms of HLH, corticosteroids with or without immunoglobulins may be sufficient to control hyperinflammation; however, initially, mild cases can deteriorate rapidly within a short time [9].

Regarding dengue-associated HLH, some have recovered spontaneously with supportive treatment only. However, in most cases, the pulse dosages of methylprednisolone or dexamethasone have been used to suppress the hyperinflammatory state. The treatment of dengue-induced HLH by intravenous immunoglobulin G is associated with a favorable outcome [28]. HLH in dengue patients responds well to the conventional treatment of HLH [10]. HLH-directed treatment with dexamethasone and etoposide showed substantially reduced mortality in potentially fatal viral infections associated with HLH [29].

Primary HLH has a near 100% fatality without adequate treatment. However, in a few international studies (HLH-94/HLH-2004), survival has increased from ~ 0 to 60% with HLH-directed treatment, including dexamethasone and cytotoxic drugs [30]. Secondary HLH is a rapidly fatal disease. Most patients die of bacterial or fungal infections due to prolonged neutropenia, multiorgan failure, or cerebral dysfunction. [8] Therefore, prompt treatment initiation is essential for patients' survival. The clinical course of HLH may be very aggressive. Sometimes initial treatment may be necessary to prevent early fatalities, even though the diagnostic workup has not been completed [23, 26]. After diagnosing HLH, our patient was started with intravenous dexamethasone for the first 2 weeks, and gradually tailed off over 8 weeks converting to oral dexamethasone. The patient had an excellent response to treatment solely with dexamethasone.

## Conclusion

This case highlights the importance of considering HLH as a potential complication when persistent fever and cytopenias present after recovering from dengue fever, especially in patients with unusual clinical features such as hepatosplenomegaly. Early recognition and prompt initiation of appropriate immunosuppressive therapy, such as intravenous dexamethasone, can lead to a successful response and good prognosis. Although different immunosuppressive agents and hemopoietic stem cell transplants have been used, our case suggests that dexamethasone can be an effective treatment option for HLH associated with dengue infection.

## Data Availability

The authors confirm that the data supporting the findings of this study are available in the article.

## Abbreviations

HIV: Human immunodeficiency virus
HLH: Haemophagocytic lymphohistiocytosis
HSCT: Haemopoietic stem cell transplant
CRP: C-reactive protein
AST: Aspartate aminotransferase
ALT: Alanine aminotransferase
DF: Dengue fever
DHF: Dengue haemorrhagic fever
DIC: Disseminated intravascular coagulation

## References

[1] Rosado FGN, Kim AS (2013). Hemophagocytic lymphohistiocytosis. Am J Clin Pathol.

[2] Gupta S, Weitzman S (2010). Primary and secondary hemophagocytic lymphohistiocytosis: clinical features, pathogenesis and therapy. Expert Rev Clin Immunol.

[3] Wiwanitkit V (2015). Haemophagocytic lymphohistiocytosis and dengue. Acta Clin Belg.

[4] Janka GE, Lehmberg K (2013). Hemophagocytic lymphohistiocytosis: pathogenesis and treatment. Hematology.

[5] Henter JI, Horne AC, Aricó M, Egeler RM, Filipovich AH, Imashuku S (2007). HLH-2004: Diagnostic and therapeutic guidelines for hemophagocytic lymphohistiocytosis. Pediatr Blood Cancer.

[6] Munshi A, Alsuraihi A, Balubaid M, Althobaiti M, Althaqafi A (2021). Dengue-induced hemophagocytic lymphohistiocytosis: a case report and literature review. Cureus.

[7] Rosado FGN, Kim AS (2013). Hemophagocytic lymphohistiocytosis. Am J Clin Pathol.

[8] Janka GE (2012). Familial and acquired hemophagocytic lymphohistiocytosis. Annu Rev Med.

[9] Janka G (2009). Hemophagocytic lymphohistiocytosis: when the immune system runs amok. Klin Padiatr.

[10] Kapdi M, Shah I (2012). Dengue and haemophagocytic lymphohistiocytosis. Scand J Infect Dis.

[11] Martina BEE, Koraka P, Osterhaus ADME (2009). Dengue virus pathogenesis: an integrated view. Clin Microbiol Rev.

[12] Ministry of Health Sri Lanka. Guidelines on Management of Dengue Fever & Dengue Haemorrhagic Fever In Adults. 2010. 1–39 p.

[13] Ustafa M (2017). Dengue fever: clinical spectrum, and management. IOSR J Dental Med Sci.

[14] Kosakai N (1968). Clinical diagnosis. Kango Kyoshitsu.

[15] Umakanth M, Suganthan N (2020). Unusual manifestations of dengue fever: a review on expanded dengue syndrome. Cureus.

[16] WHO. Laboratory diagnosis and diagnostic tests-Dengue-NCBI Bookshelf. 2009.

[17] Teparrukkul P, Hantrakun V, Day NPJ, West TE, Limmathurotsakul D (2017). Management and outcomes of severe dengue patients presenting with sepsis in a tropical country. PLoS ONE.

[18] Syue LS, Tang HJ, Hung YP, Chen PL, Li CW, Li MC (2019). Bloodstream infections in hospitalized adults with dengue fever: Clinical characteristics and recommended empirical therapy. J Microbiol Immunol Infect.

[19] Gulati S, Maheshwari A (2007). Atypical manifestations of dengue. Trop Med Int Health.

[20] Deane S, Selmi C, Teuber SS, Gershwin ME (2010). Macrophage activation syndrome in autoimmune disease. Int Arch Allergy Immunol.

[21] La RP, Horne AC, Hines M, Greenwood TVB, Machowicz R, Berliner N (2019). Recommendations for the management of hemophagocytic lymphohistiocytosis in adults. Blood.

[22] Grom AA (2004). Natural killer cell dysfunction: a common pathway in systemic-onset juvenile rheumatoid arthritis, macrophage activation syndrome, and hemophagocytic lymphohistiocytosis?. Arthritis Rheum.

[23] Aricò M, Danesino C, Pende D, Moretta L (2001). Pathogenesis of haemophagocytic lymphohistiocytosis. Br J Haematol.

[24] Jordan MB, Hildeman D, Kappler J, Marrack P (2004). An animal model of hemophagocytic lymphohistiocytosis (HLH): CD8+ T cells and interferon gamma are essential for the disorder. Blood.

[25] Jordan MB, Allen CE, Greenberg J, Henry M, Hermiston ML, Kumar A (2019). Challenges in the diagnosis of hemophagocytic lymphohistiocytosis: recommendations from the North American Consortium for Histiocytosis (NACHO) Pediatric Blood and Cancer.

[26] De Koninck AS, Dierick J, Steyaert S, Taelman P (2014). Hemophagocytic lymphohistiocytosis and dengue infection: rare case report. Acta Clin Belg.

[27] Khurram M, Faheem M, Umar M, Yasin A, Qayyum W, Ashraf A (2015). Hemophagocytic lymphohistiocytosis complicating dengue and *Plasmodium vivax* coinfection. Case Rep Med.

[28] Ray U, Dutta S, Mondal S, Bandyopadhyay S (2017). Severe dengue due to secondary hemophagocytic lymphohistiocytosis: a case study. IDCases.

[29] Gao C, Cai Y, Zhang K, Zhou L, Zhang Y, Zhang X (2020). Association of hypertension and antihypertensive treatment with COVID-19 mortality: a retrospective observational study. Eur Heart J.

[30] Kan FK, Tan CC, von Bahr GT, Khalid KE, Supramaniam P, Myrberg IH (2020). Dengue infection complicated by hemophagocytic lymphohistiocytosis: experiences from 180 patients with severe dengue. Clin Infect Dis.

